# Non-O1/Non-O139 *Vibrio cholerae*—An Underestimated Foodborne Pathogen? An Overview of Its Virulence Genes and Regulatory Systems Involved in Pathogenesis

**DOI:** 10.3390/microorganisms12040818

**Published:** 2024-04-18

**Authors:** Quantao Zhang, Thomas Alter, Susanne Fleischmann

**Affiliations:** Institute of Food Safety and Food Hygiene, School of Veterinary Medicine, Freie Universität Berlin, Königsweg 69, 14163 Berlin, Germany; quantao.zhang@fu-berlin.de (Q.Z.); thomas.alter@fu-berlin.de (T.A.)

**Keywords:** non-O1/non-O139 *Vibrio cholerae*, diarrheal disease, infection pathway, virulence genes, regulatory systems

## Abstract

In recent years, the number of foodborne infections with non-O1 and non-O139 *Vibrio cholerae* (NOVC) has increased worldwide. These have ranged from sporadic infection cases to localized outbreaks. The majority of case reports describe self-limiting gastroenteritis. However, severe gastroenteritis and even cholera-like symptoms have also been described. All reported diarrheal cases can be traced back to the consumption of contaminated seafood. As climate change alters the habitats and distribution patterns of aquatic bacteria, there is a possibility that the number of infections and outbreaks caused by *Vibrio* spp. will further increase, especially in countries where raw or undercooked seafood is consumed or clean drinking water is lacking. Against this background, this review article focuses on a possible infection pathway and how NOVC can survive in the human host after oral ingestion, colonize intestinal epithelial cells, express virulence factors causing diarrhea, and is excreted by the human host to return to the environment.

## 1. Introduction

*Vibrio* (*V.*) *cholerae* is mainly known as the causative agent of the endemic and epidemic diarrheal disease cholera. However, *V. cholerae* is a globally distributed aquatic commensal that has been classified into more than 200 serogroups. Only two serogroups, O1 and O139, have the ability to cause pandemic cholera outbreaks. Since 1961, the *V. cholerae* serotype O1 biotype El Tor has been the predominant strain in the seventh pandemic, and since 1992, the *V. cholerae* serotype O139 has been the predominant strain in the eighth pandemic. Both pandemics are still ongoing today [1,2]. The World Health Organization (WHO) reported outbreaks of cholera in 30 countries in Asia, Africa, and America between 1 January and 15 December 2023, with over 667,000 cases and 4000 deaths [3].

Non-O1 and non-O139 *V. cholerae* (NOVC) serogroups are less in the focus of public health interest compared to O1 and O139 *V. cholerae* as they cause single-case disease or even localized outbreaks with milder and often self-limiting symptoms. In fact, one or both of the main virulence factors, cholera toxin (CT) and toxin-coregulated pilus (TCP), are missing in their genomes. Nevertheless, NOVCs are playing an increasingly important role in public health worldwide. Several studies have shown that the number of infections and outbreaks caused by NOVC has increased over time, being positively correlated with the progressive rise in seawater temperatures [4,5,6,7]. This is promoted by the anthropization of coastal regions, the increasing global trade of seafood, the trend towards the consumption of raw seafood (e.g., oysters and sushi), and the increasing number of immunocompromised people, especially older people with pre-existing diseases [7,8]. Particularly people with a compromised immune system can suffer from severe diarrhea with cholera-like symptoms. Bacteremia can also be caused by an orally acquired infection via the infiltration of NOVC in the bloodstream through the portal vein and intestinal lymphatic system [8,9,10]. Individual infection cases that could be clearly attributed to the consumption of contaminated seafood have been described in Spain [11], Italy [12], Portugal [13], India [14], Australia [15], the USA [16], and Iran [17]. Localized NOVC outbreaks have been reported in India and Thailand in the past [18,19,20,21,22,23]. Meanwhile, NOVC outbreaks have also been described in the USA [24,25], China [26,27], and Chile [28] which have also been linked to seafood consumption.

Octavia et al., 2013, pointed out that a combination of virulence factors in the genome of clinical NOVC is a prerequisite for a successful infection process [29]. The combined virulence factors identified in the genomes of NOVC isolated from the above-mentioned infection cases and local outbreaks are the *Vibrio* pathogenicity islands VSP-2 and VPI-2, genomic islands (GI) encoding type III (T3SS) and type VI secretion systems (T6SS), enterotoxins (RtxA and Stn), and the hemolysin HlyA. We were able to show that genes encoding these virulence factors are also present in NOVC isolated from seafood and the environment in previous studies [30,31]. Further investigations showed that other virulence genes are also present in the NOVC genomes which could also play a role in the infection process, such as *hapA* for hemagglutinin protease; *mshA* for mannose sensitive hemagglutinin; and *frhA*, *gbpA*, and *mam7* for non-specific adhesins [30].

In addition to the presence of virulence factors, genes involved in host adaption and colonization are also required in the pathogenicity process. Before a successful infection, pathogens need to survive the host defense system such as acidic pH values in the stomach, anti-microbial peptides, reactive oxygen species (ROS), and an already predominant gut microbiota [32]. Mucosal penetration and epithelial cell attachment in the small intestine are also necessary for the final infection and proliferation of the bacteria [33]. *V. cholerae* has evolved a complex regulation system to ensure proper arrangement of various effective factors throughout the infection inside a human host, such as the quorum sensing system, two-component system, histone-like nucleoid structuring protein (Hns), small molecule signals (c-di-GMP), biofilm promotor and motility repressor modulation, and wide spectrum regulator (cAMP-CRP) [34].

**Figure 1:**
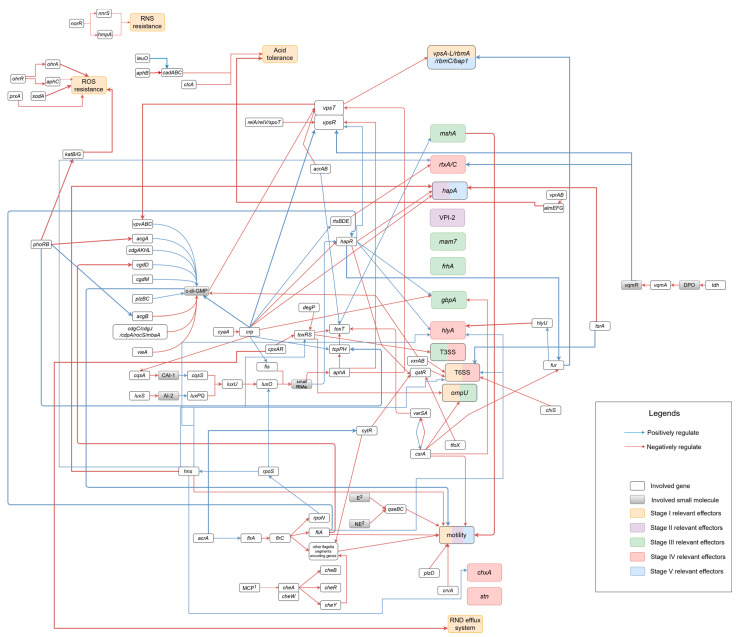
The map of virulence-associated genes and regulatory systems in NOVC: positive relationships are labeled with red arrows and negative relationships are labeled with blue arrows. The whole infection procedure is separated into five stages. Stage 1: survival in host gastrointestinal tract (in orange); stage 2: localization and penetration of the mucus layer in the small intestine (in purple); stage 3: intestinal epithelial cell colonization (in green); stage 4: virulence gene expression (in red); stage 5: detachment from the epithelial cells to return in the environment (in blue). The detailed information is shown in Table S1 in the Supplementary Files.

Thus far, there are several comprehensive overviews about the virulence-associated genes in both O1/O139 *V. cholerae* and NOVCs [35,36]. Nevertheless, it should be noted that the relationship between virulence factors and a resulting infection is complex, and an interaction network rather than individual virulence factors must be considered at this point. The previous findings on virulence-associated genes and their interaction with other genetic features involved in the infection process will be discussed in this review article. Furthermore, a genetic model of a theoretical infection caused by NOVC inspired by the Kyoto Encyclopedia of Genes and Genomes (KEGG) mapping tool [32,37,38,39] was developed (see Figure 1). The whole infection workflow was divided into five stages as follows. Stage 1: survival in host gastrointestinal tract; stage 2: localization and penetration of the mucus layer in the small intestine; stage 3: intestinal epithelial cell colonization; stage 4: virulence gene expression; stage 5: detachment from the epithelial cells to return in the environment.

## 2. Stage 1: Survival in the Gastrointestinal Tract

After oral ingestion, pathogenic bacteria will encounter a set of host-derived defense systems, including chemical and biological barriers, when entering the stomach and arriving at the small intestine. Therefore, various genes involved in adaptation processes as a response to these conditions can be found in NOVCs to ensure that they reach the small intestine to interact with epithelial cells [40,41]. The stage 1 section therefore describes the adaptation to low pH values in the stomach [41,42]; the adaptation to reactive nitrogen and oxygen species in the stomach [43,44]; changes in porin channel size to prevent the diffusion of harmful molecules into the bacterial cell such as bile salts from the gallbladder in the duodenum [45]; efflux pumps to displace harmful molecules such as bile in the duodenum and antimicrobial peptides in the small intestine [46]; the formation of protective biofilms to protect bacteria against antimicrobial substances from the stomach, duodenum, and the small intestine; and the T6SS to compete with the predominant gut microbiome [47]. All mechanisms and genes involved in stage 1 are shown in orange in Figure 1.

### 2.1. Acid Tolerance Response

A common feature of diarrheal pathogens is the acid tolerance response (ATR), necessary to survive the acidic pH environment in the stomach, which is a prerequisite for subsequent successful intestinal colonization [42]. In *V. cholerae*, the *cadABC* operon first described in *Escherichia* (*E.*) *coli* is important for protecting the bacteria from acid hydrolysis [40]. The genetic presence of the *cadABC* operon was identified in 90% of NOVC isolated from seafood and the environment in our previous studies, with genetic identities over 90% compared to the *V. cholerae* O1 El Tor biotype, suggesting a fully functional *cadABC* operon [30,31]. In particular, the *cadA* gene encodes a lysine decarboxylase that binds protons through the production of cadaverine and carbon dioxide. Finally, this antiporter system transfers protons out of the bacterial cell and neutralizes the pH value [40]. Kovacikova et al., 2010, mentioned that *cadC,* the regulator of the *cadABC* operon, can be directly activated by *aphB* encoding a cytoplasmic DNA-binding protein which will be upregulated during acid stress [48]. Additionally, the expression of *clcA*, a hydrochloric acid transporter, regulated by *aphB*, plays a role in neutralizing the pH value in the bacterial cell [49].

### 2.2. Adaptation to Reactive Nitrogen and Oxygen Species

In the stomach, nitrite from food and saliva that is exposed to the acidic milieu results in acidified nitrite, which can be reduced by reactive nitrogen species (RNS) to antimicrobially active nitric oxide. By the detoxification of RNS, the expression of the genes *nnrS* and *hmpA* plays an important role in *V. cholerae*, and these genes were also identified in NOVCs from seafood and the environment [30,31]. Both genes encode enzymes that are capable of destroying nitric oxide. It is assumed that the regulator for both genes is *norR*, although this regulator is not stimulated by nitric oxide [43].

In diarrheal diseases, the level of reactive oxygen species (ROS) in the host gastrointestinal tract increases, resulting in damage to the bacterial cell structure as an immune defense. In *V. cholerae*, genes with ROS resistance activity have been identified as part of ROS removal. Superoxide dismutases such as manganese-binding SodA, for example, convert superoxide into hydrogen peroxide and oxygen. Catalases such as KatB and KatG later detoxify peroxides into water and oxygen [50,51]. The organic hydroperoxidase OhrA and preoxiredoxins such as PrxA and AphC cleave organic (alkyl) hydroperoxides [52,53]. Two homologs of *ohrR*, the gene for hydrogen peroxide resistance in *E. coli*, were found as well in *V. cholerae,* namely *oxyR*1 and *oxyR*2, which have a modulating function on *prxA* and *aphC*, respectively [52].

### 2.3. Resistance Nodulation Division (RND) Efflux Pump

After passing through the stomach, pathogenic bacteria in the small intestine must resist against host-derived bile salts, organic acids, and antimicrobial peptides. The main systems which help the bacteria to pump numerous poisonous compounds out of the cell are efflux pumps [54]. The RND efflux pump is a multi-functional unit in both O1/O139 *V. cholerae* and NOVC [55], encoded by a vex gene cluster (*vexAB*, *vexCD*, *vexEF*, *vexGH*, *vexIJK*, and *vexLM*) and a shared outer membrane porin encoded by *tolC*. Using an infant mouse model, small intestine colonization deficiency was found in different RND mutants [46,56].

### 2.4. Outer Membrane Protein (OMP)

Another response to toxic components such as bile salts is the alteration of porins in the bacterial cell membrane. In *V. cholerae*, expression and upregulation of *ompU* take place when bile salts are present. OmpU is widely present in NOVC [9,57,58] and should have the same response to the presence of bile. Due to its smaller channel size which prevents the influx of bile salts into the bacterial cell, the porin OmpU will be replaced instead of a larger channel porin such as OmpT [45,59].

### 2.5. Biofilm Formation

Biofilms provide a continuous protective cover around bacterial cells against multiple harmful components and play an important role in environmental adaptation and survival in the host [39]. In *V. cholerae*, *Vibrio* polysaccharides encoded by *vpsA* to *vpsK* and other biofilm-forming proteins encoded by the genes *rbmA*, *rbmC*, and *bap1* are the main structure components that build a stable biofilm [60,61], while *vpsR* and *vpsT* serve as transcriptional activators [62,63]. Another small molecule signal, c-di-GMP, also has a positive effect on biofilm formation through the upregulation of *vpsR* and *vpsT* [64]. The intracellular concentration of c-di-GMP can be increased in the presence of bile [65]. The presence of *vpsR* in NOVC was confirmed by Dua et al. (2018) [66], and variations in VpsR between O1/O139 *V. cholerae* and NOVC were identified in our previous studies, including point mutation and gene fragment deletions [30]. However, 99% of NOVCs could form stable biofilms in our previous studies, and all the biofilm-relevant genes were present in NOVCs [30,31].

### 2.6. Type IV Secretion System (T6SS)

Entering the small intestine, the T6SS plays an important role in competition between microorganisms, so that the distribution of commensals in the intestine is altered [47,67]. Therefore, it might act in stage 1 as well as in stage 4. In our previous studies, all NOVCs contained the T6SS [30,31]. In the *V. cholerae* strain O1 C6706, the T6SS is repressed at low cell density by quorum sensing (QS) molecules [68]. In contrast, N-acetyl glucosamine (GlcNac) can be sensed by the O1 *V. cholerae* serotype, which leads to *tfoX* (a major regulator of T6SS) expression followed by T6SS activation [69]. The regulation network of NOVCs is complex and not fully explored, but these regulators might have similar effects in NOVCs [70]. Three regulatory genes, *hapR*, *tfoX*, and *cytR*, achieve their T6SS regulation through the QS- and TfoX-dependent regulator (QstR) [34].

## 3. Stage 2: Localization and Penetration of the Mucus Layer in the Small Intestine

To cause diarrhea, *V. cholerae* need to reach the small intestinal epithelial cells to penetrate them. However, the intestinal epithelium is covered by a mucus layer (approximately 150 μm thick), making the ability to penetrate mucus important [32]. Motility is therefore necessary and responsible for a targeted direction [71], while the contribution of chemotaxis remains controversial [72,73]. In contrast to O1/O139 *V. cholerae* serotypes, whose fitness is supported by genetic features on *Vibrio* seventh pathogenicity islands 1 and 2 (VSP-1 and VSP-2), the movement of NOVC through the mucosa could be supported by the hemagglutinin protease and neuraminidase, which act as mucinases and are encoded on *Vibrio* pathogenicity island 2 (VPI-2) [1,74,75]. In addition, environmental NOVCs isolated from food and water sources carry not only the pathogenicity island VPI-2 but also the pathogenicity island VSP-2 in their genome [30,31]. All mechanisms and genes involved in stage 2 are shown in purple in Figure 1.

### 3.1. Motility via Flagella

The motility-related genes in NOVC were detected through Gene Oncology analysis and the KEGG pathway, which indicate the similar function of motility between NOVC and O1/O139 *V. cholerae* as these databases are mainly built based on research on O1/O139 *V. cholerae* [76]. All the motility-associated genes were identified to 100% in NOVC according to our previous studies [30,31]. As a highly motile bacterium, the driving force of *V. cholerae* is provided by a single polar flagellum. Motility is also functional in host environment adaptation, including nutrient acquisition and toxic component avoidance [77]. The flagellar motility of *V. cholerae* is important to move the bacteria through the mucus layer [78]. The structure of the flagellum and its four-hierarchy regulatory system was already described by Syed et al. (2009) [79]. The whole flagella system is regulated by sigma factor 54 FlrA, the downstream activator FlrC, and the alternative sigma 28 factor FliA [79]. The motility of *V. cholerae* also declined due to c-di-GMP. Furthermore, several genes with motility regulation activity were reported. The multifunctional regulation gene *csrA* could upregulate *flrC* [80]. Besides, under a high-speed microscope, *arcA*/*cytR* and the O-antigen synthesis gene *cmd* were found to promote motility with an unclear mechanism [81].

### 3.2. Chemotaxis

The chemotaxis system can recognize chemical signals and regulate the motility and swimming behavior of *V. cholerae*. At first, a common chemotaxis model of *E. coli* was identified and subsequently applied in a chemotaxis study of *V. cholerae* [82] including methyl-accepting chemotaxis proteins (MCPs) encoded by *cheW*, *cheA*, *cheY*, *cheR*, and *cheB*. As the chemotaxis system in NOVC has not been explored and the chemotaxis-related genes in O1/O139 *V. cholerae* were identified in several NOVCs, we suspected that the chemotaxis system in NOVCs might play a similar role. In our previous study, we confirmed the presence of the genes *cheA*, *cheY*, and *cheR* in all analyzed NOVCs, while *cheW* and *cheR* were present in 32% of the strains [30]. Transmitted signals can be caught by the cytoplasmic linker protein *cheW* and transmitted to the two-component system *cheA*/*cheY*. Phosphate-activated *cheY* binds to the flagella motor and causes a reverse rotation direction, from left to right [72]. The genes *cheR* and *cheB* play a role in the transfer of methyl groups, which contributes to adaptation to a stable background level of attractants [82]. On the other hand, *V. cholerae* (both O1/O139 and NOVC) have a far more complex chemotaxis system than *E. coli*, with 68 related ORFs categorized into three clusters. Among those, cluster II seems to play a similar role in *E. coli* [83]. Later research reported that cluster I components are assembled into the supramolecular signaling complex in response to reduced cellular energy states, raising the possibility that the cluster I complex plays a role in sensing and signaling under microaerobic environments, such as in the host intestine [84]. The general stress regulator RpoS and autoinducer 1 in quorum sensing could regulate the expression of cluster III [85].

### 3.3. Vibrio Pathogenicity Island 2 (VPI-2)

VPI-2 (located between vc1758 and vc1809) was identified in O1/O139 *V. cholerae* and NOVCs. However, Jermyn and Boyd (2005), Haley et al. (2014), and Takahashi et al. (2021) studied the genetic variation of VPI-2 in NOVCs and showed that NOVCs could harbor an incomplete VPI-2 compared to O1/O139 [74,86,87]. This variation might result from the horizontal gene transfer of VPI-2 from the ancestors *V. mimicus* and O1/O139 *V. cholerae* [74]. VPI-2 contains the neuraminidase-encoding gene *nanH* [88] which plays a role in altering mucus structure by cleaving sialic acid groups (GM1 gangliosides) on the epithelial cell surface. Further studies on VPI-2 revealed that *nanA*, *nanE*, *nanK*, and *nagA1*, which are also localized on VPI-2, can catalyze the metabolism of N-acetylneuraminic acid, which is a component of mucin [6]. All of these functional genes in VPI-2 were identified in 33% of NOVCs isolated from seafood and the environment [30,31].

Vertebrate hosts could limit the zinc level for bacteria as a defense strategy. Zinc deficiency activates *Vibrio* energy taxis system A (VerA), which is also encoded on the pathogenicity island VSP-2. In addition, VerA could trigger the expression of *aerB* transcribing a methyl-accepting chemotaxis protein, which could bind *cheW* and affect the flagellum rotation and motility [89,90].

### 3.4. Hemagglutinin Protease HapA

The hemagglutinin protease HapA, encoded by *hapA*, is suggested to be responsible for altering the mucus layer and playing a role in mucus layer penetration during initial infection for both O1/O139 *V. cholerae* and NOVC [91,92].

## 4. Stage 3: Intestinal Epithelial Cell Colonization

After the localization of intestinal epithelial cells, *V. cholerae* must attach to their surface, whereby the type IV pili and the T3SS play a crucial role [93,94]. Subsequently, non-specific adhesins can be secreted via these systems [32]. In contrast to O1 and O139 *V. cholerae*, the T3SS plays an important role for NOVC in attachment and colonization when TCP is not present in the genome [94]. All mechanisms and genes involved in stage 3 are shown in green in Figure 1.

### 4.1. Type IV Pili

Type IV pili, encoded by *mshA*, play a role in the braking and anchoring function of *V. cholerae* during the landing process on the epithelial cell surface [95]. In addition, MshA pili cause an irreversible attachment and microcolony formation [93]. The presence of *mshA* in 27% of NOVCs was confirmed in our previous studies [30,31]. At the beginning stage after landing on the epithelial cell surface, several transient non-specific adhesins were secreted to bind the component of small intestine epithelial cells, including multivalent adhesion molecule 7 (*mam7*, binding with fibronectin and phosphatidic acid), GlcNAc binding protein A (encoded by *gbpA*), and flagellum-regulated hemagglutinin A (encoded by *frhA*, binding calcium) [79,96,97]. The presence rates of *mam7*, *gbpA*, and *frhA* in NOVCs were detected as 100%, 94%, and 22%, respectively, in our previous studies [30,31]. Sperandio et al., 1995, stated that a potential adherence factor to epithelial cells could be the outer membrane protein U (OmpU) [98]. This finding is supported by Potapova et al., 2024, who also mentioned that OmpU could also regulate the biofilm matrix assembly [99].

### 4.2. Type III Secretion System (T3SS)

The T3SS is suggested to have an important role in the colonization of intestinal epithelial cells by NOVCs when TCP is absent. Dziejman et al., 2005, showed using a rabbit and mouse model that the TCP-negative NOVC strain AM-19226 could colonize the intestinal epithelial cell surface through the T3SS [94]. The whole island contains 47 ORFs from A33_1660 to A33_1706. However, an exact mechanism of the T3SS in colonization has not yet been fully identified, although possible functions of several effectors have been addressed: VopF (A33_1696) and VopM (A33_1684) are two effectors in the core region with actin alteration activities, which could disrupt the cell structure and contribute to colonization [100]. VopM can bind F-actin and also plays an important role in colonization by remodeling the intestinal brush border, which facilitates bacterial adhesion [101]. The colonization activity of VopX (A33_1663) in AM-19226 was also stated by Alam et al. [102], and a contradictive result was reported by Chaand et al. [103]. Meanwhile, the T3SS is important for toxicity and toxin transfer; therefore, this part is explained further in stage 4.

## 5. Stage 4: Virulence Factor Expression

In contrast to the *V. cholerae* serovars O1 and O139, which express cholera toxin (CTX) and its accessory toxins within the CTX phage, various toxins can be produced by NOVCs after colonization of the small intestine. Currently, four secreted proteins with direct toxic effects shown on cell lines and in animal models have been identified: the hemolysin HlyA, repeats-in-toxin (RTX), heat-stable enterotoxin (ST), and cholix toxin (ChxA) [104,105,106,107]. The expression of these toxins leads to an alteration in the morphology of epithelial cells, cell damage, and subsequently to the death of the cells [35]. Similar to *V. cholerae* serotypes O1 and O139, whose virulence is supported by genetic features on VPI-1 and VPI-2 [1], VPI-2 was also identified in NOVC environmental isolates from food and water [30,31]. In addition to the toxin genes, the T3SS also plays an important role for NOVC toxicity by secreting virulence factors from the bacteria to the host cells when TCP is missing in the genome [94]. All mechanisms and genes involved in stage 4 are shown in red in Figure 1.

### 5.1. Toxin Expression

The hemolysin HlyA (also called *V. cholerae* cytolysin, VCC) could both lyse erythrocytes and form beta barrel pores on epithelial cells [104], followed by cytoskeleton damage, cell lysis, and diarrhea. The iron extracted from the cells in this way serves as a nutrient supplier for NOVCs [108,109]. The transcription of *hlyA* in *V. cholerae* is regulated by QS molecules, which regulate *hlyU*, resulting in the highest transcription of *hlyA* in the early mid-logarithmic growth phase [110].

Repeats-in-toxin (RTX) is a large protein (around 3500 to 5300 amino acids) widely present in many bacteria which could cause tight junction loss in lung and intestinal epithelial cells [111]. The in vivo toxicity of RTX in *hlyA*-harboring *V. cholerae* tends to present as innate immune evasion rather than diarrhea [105,112]. Three major functional units of RTX were found in *V. cholerae* O1 El Tor N16961. The actin cross-linking domain (ACD) is responsible for cytoskeleton disruption, the Rho GTPase-inactivation domain (RID) for cell rounding, and the alpha/beta hydrolase domain (ABH) for autophagic/endosomal trafficking inhibition. An additional cysteine protease domain is responsible for effectors’ autoprocessing and distribution. The combination of RID and ABH could reduce the inflammatory response caused by ACD [113]. Compared to the El Tor O1 serogroup, NOVCs have more variations in their RTX domain [114]. A nucleotide cluster with five ORFs is responsible for the coding of RTX: the encoding toxin gene *rtxA*, the activator gene *rtxC*, and the associated ABC transportation gene cluster *rtxBDE* [113]. The whole RTX complex was identified in 61% of NOVCs in our previous studies [30,31].

Heat-stable enterotoxin (ST, encoded by *stn*) is a known toxin in *E. coli* and was also identified in the genome of NOVC [106,115]. The in vivo toxicity was attributed to fluid accumulation in mouse intestine [106]. The toxin consists of two domains, STa and STb. STa leads to anion secretion and calcium absorption, while STb could decrease the expression of the tight junction proteins ZO-1 and occludin [116]. 

The cholix toxin ChxA interacts with prohibitin and could therefore cause mitochondrial dysfunction and cytoskeletal remodeling. It is able to bind the lipoprotein receptors of the intestinal epithelial cells and inhibit protein synthesis by ADP-ribosylation. The in vivo toxicity presented as liver damage and final death through mouse assay [107,117]. Tangestani et al., 2020, also confirmed the presence of cholix toxin in NOVC [17].

### 5.2. Type III Secretion System (T3SS)

The T3SS plays an important role in NOVC after the colonization of intestinal epithelial cells in toxicity. Dziejman et al., 2005, suggested using a mouse model that the T3SS-positive NOVC strain AM-19226 causes mouse death in contrast to a T3SS-negative mutant strain [94]. The protein VopF contains three WASP homology 2 (WH2) actin-binding domains, which could remodel the actin cytoskeleton in eukaryotic host cells. The actin polymerization disorder triggered by VopF is essential for T3SS-mediated intestinal cell damage in AM-19226 [118]. The mechanism might be that VopF could induce cortical actin depolymerization and aberrant localization of the tight junction protein ZO-1, resulting in loosening of the tight junction between intestinal epithelial cells and causing diarrhea [119]. However, Miller et al., 2016, observed that cell death and disruption of the tight junction are independent of VopF [120]. It has been suggested that VopE (A33_1662) impairs mitochondrial dynamics and stimulates the innate immune pathway [121]. Furthermore, the in vivo toxicity of VopE was verified in an infant rabbit and mouse model [122]. The regulators VttRA and VttRB, which show homology with ToxR, can control T3SS activity both during colonization and pathogenesis [123].

#### Bacteremia Caused by NOVCs

When NOVCs enter and colonize the small intestine (as described in stages 1 to 3), they could enter the bloodstream through the portal vein and the intestinal lymphatic system [8,9]. The immune system, macrophages, and specific antibodies are involved in the blood defense system, indicating that genes for immune modulation are important for NOVCs to cause blood infections [124,125]. Hemolytic properties, such as the presence of HlyA, suggest their ability to enter the bloodstream and lyse erythrocytes [104]. RTX could protect NOVCs from neutrophil-dependent clearance [105]. Biofilms could protect NOVCs from leukocytes of the human immune system. Additionally, NOVCs can form biofilms on the eukaryotic cell surface, causing a concentration of MshA and HapA, which could increase the local hemolysin level lysing immune cells [126].

## 6. Stage 5: Detachment from the Epithelial Cells

At the end of the infection cycle, NOVCs return to the environment through watery to bloody diarrhea. The symptoms of infection can be closely similar to those of the cholera caused by the serotypes O1 and O139. The starvation/stationary phase alternative sigma factor RpoS positively controls the expression of HapR, a gene involved in flagella assembly and chemotaxis. This enables the detachment and migration of NOVCs from the epithelial cells into the lumen of the intestine [127]. After the activation by RpoS, the hemagglutinin protease HapA encoded by *hapA* is responsible for detachment from intestinal epithelial cells [75]. Apart from the mucinase activity, HapA could degrade GbpA, the non-specific adhesin in colonization [128]. Furthermore, a set of potential biofilm degradation genes were also identified by Bridges et al., 2020 [129]. These genes include ribosome-associated GTPase encoded by *bipA*; c-di-GMP phosphodiesterases encoded by *cdgG*, *cdgI*, *rocS*, and *mbaA*; a polyamine transporter encoded by *potD*1; a peptidase encoded by *lapG*; a polysaccharide lyase encoded by *rbmB*; and a chemotaxis regulator encoded by *cheY*3. All genes were controlled by the two-component system *dbfS*/*dbfR* [129]. All the mentioned mechanisms and genes involved in stage 5 are shown in blue in Figure 1.

## 7. Multifunctional Regulation System

NOVCs have evolved several regulators to ensure the expression of genes that lead to successful colonization of the intestinal tract. In addition, NOVCs have evolved a number of adaptive mechanisms to adapt to both the environment and the human host as well as to the transition between host and environment [34]. One such multifunctional regulation system is quorum sensing. By cell-to-cell communication, NOVC is able to adjust the cell density. Three QS pathways through different chemical signals have evolved: cholera autoinducer 1 (CAI-1), autoinducer 2 (AI-2), and 3,5-dimethylpyrazin-2-ol (DPO) [130,131,132]. The downstream genes of CAI-1 and AI-2 are *cqsS* and *luxPQ*, respectively, followed by *luxO*, *aphA*, and *hapR* (Figure 1) [133]. DPO is the third QS signal mechanism, which can be sensed by *vqmA* [134], followed by the release of the small molecule *vqmR* to downregulate *rtxA* and *vpsR* [135,136].

Two-component systems are another set of regulators with a wide range of functions. Within the two-component system *varS*/*varA*, a receptor for QS and environmental signals represents, together with its downstream gene *csrA*, a multifunctional regulator in biofilm regulation, iron metabolism, virulence gene expression, and motility [80,137,138]. Another two-component system is *vprA*/*vprB*, which is involved in polymyxin and bile resistance [139], which also demonstrated a mutant strain showing colonization failure in host intestine in a mouse model. The gene set *vxrA*/*vxrB* could upregulate T6SS expression and biofilm formation [140,141]. The expression of *phoR*/*phoB* is activated by phosphate limitation, followed by repression of biofilm-related genes and upregulation of motility [142]. The *qseB*/*qseC* gene set is a receptor of the hormones epinephrine and norepinephrine and could affect bacteria motility through triggering *pomB* expression [143,144]. To adapt to oxygen-poor conditions, *acrB*/*acrA* could upregulate *toxT* and enhance biofilm formation and ROS resistance [145]. *chiS* is both the monitor and regulator of (ClcNac)2, which is important for intestinal epithelial cell adherence and gut fluid accumulation [146,147].

Two global regulators are histone-like nucleoid structuring protein (HNS) and cyclic AMP-activated global transcriptional regulator (cAMP-CRP). HNS acts as a mediator at the late stage of infection with a repressive effect on a large number of virulence-associated genes such as hemolysin *hlyA* [148], repeat-in-toxin *rtxA* [149], *Vibrio* polysaccharide *vps* [51], and the T6SS [150], while it could also promote motility and the detachment-dependent protein HapA [151]. The global regulator CRP is the receptor of cAMP, the secondary messenger, and acts as a key regulator of many genes in response to lifestyle changes, including the genes *rtxBDE* and *hlyA* [152]. Moreover, CRP represses biofilm formation by repressing the genes *vpsR*, *vpsT*, and *vpsL* and, at the same time, activating the high cell density regulator HapR [153].

## 8. Schematic Infection Pathway of NOVC

Based on the research mentioned above, a schematic map of virulence-associated genes in NOVC was established and is summarized in Figure 1.

## 9. Conclusions

As this review article shows, the oral infection of human hosts by pathogenic NOVC is a complex process that depends on the infectivity of the bacterial cells and their ability to survive the harsh conditions in the host until they return to the environment.

It is known that the virulence profile of NOVCs varies, but among them, there are strains expressing all or most of the virulence genes and regulatory systems described in this review article, possibly leading to a pathogenesis ranging from self-limiting diarrheal diseases to cholera-like symptoms and/or bacteremia. Thus, this review article provides an overview of a variety of virulence-associated genes and regulatory systems supporting the understanding of how and why foodborne NOVCs can cause infections.

## Supplementary Materials

The following supporting information can be downloaded at: https://www.mdpi.com/article/10.3390/microorganisms12040818/s1, Table S1: Additional information of virulence-associated genes in NOVC.
